# Construction and Analysis of a ceRNA Network Reveals Potential Prognostic Markers in Colorectal Cancer

**DOI:** 10.3389/fgene.2020.00418

**Published:** 2020-05-08

**Authors:** Li Guo, Guowei Yang, Yihao Kang, Sunjing Li, Rui Duan, Lulu Shen, Wenwen Jiang, Bowen Qian, Zibo Yin, Tingming Liang

**Affiliations:** ^1^Department of Bioinformatics, Smart Health Big Data Analysis and Location Services Engineering Lab of Jiangsu Province, School of Geographic and Biologic Information, Nanjing University of Posts and Telecommunications, Nanjing, China; ^2^School of Life Science, Jiangsu Key Laboratory for Molecular and Medical Biotechnology, Nanjing Normal University, Nanjing, China; ^3^Changzhou Institute of Innovation & Development, Nanjing Normal University, Nanjing, China

**Keywords:** colorectal cancer, competing endogenous RNA, ceRNA network, prognostic marker, long noncoding RNA, miRNA

## Abstract

Colorectal cancer (CRC) is one of the leading causes of cancer-related deaths worldwide and is derived from an accumulation of genetic and epigenetic changes. This study explored potential prognostic markers in CRC *via* the construction and in-depth analysis of a competing endogenous RNA (ceRNA) network, which was generated through a three-step process. First, we screened candidate hub genes in CRC as the primary gene markers to survey their related regulatory non-coding RNAs, miRNAs. Second, the interacting miRNAs were used to search for associated lncRNAs. Thus, candidate RNAs were first constructed into ceRNA networks based on close associations with miRNAs. Further analysis at the isomiR level was also performed for each miRNA locus to understand the detailed expression patterns of the multiple variants. Finally, RNAs were performed an in-depth analysis of expression correlations, which contributed to further screening and validation of potential RNAs with close correlations to each other. Using this approach, nine hub genes, 13 related miRNAs, and 29 candidate lncRNAs were collected and used to construct the ceRNA network. Further in-depth analysis identified the MFAP5-miR-200b-3p-AC005154.6 axis as a potential prognostic marker in CRC. MFAP5 and miR-200b-3p have previously been reported to play important roles in tumorigenesis. These RNAs showed potential prognostic values, and the combination of them may have more sensitivity than using them alone. In conclusion, MFAP5, miR-200b-3p, and AC005154.6 may have potential prognostic value in CRC and may provide a prognostic reference for this patient population.

## Highlights

– Nine hub genes were screened in colorectal cancer, and then these were used to survey related microRNAs.
– Long non-coding RNAs were screened based on associations with these miRNAs. The full set of candidate RNAs were constructed into a competing endogenous RNA network.
– Further in-depth analysis verified the MFAP5-miR-200b-3p-AC005154.6 axis as a potential prognostic marker for colorectal cancer.

## Introduction

Colorectal cancer is one of the most common malignancies. Although significant advancements have been made in the early diagnosis and treatment of CRC, there remains an increased risk of cancer-related death from CRC in the United States (Rahman et al., 2015; Callahan et al., 2019). CRC also has a high incidence and is the second leading cause of cancer-related deaths in European countries (Altobelli et al., 2014). The annual rate of new CRC diagnoses is increasing worldwide (Ferlay et al., 2015), but most patients can be treated at early stages (Pawa et al., 2011). Both environmental and genetic factors influence the occurrence and development of cancer. For example, the pathological progression of CRC is a multistep processes that is caused by the accumulation of genetic alterations, primarily gene mutations and epigenetic changes (Worthley et al., 2007). Chronic infections and the ensuing inflammation also contribute to pathophysiological processes, tumor initiation, and progression. Additionally, inflammation is a crucial hallmark of cancer that is caused by multiple factors (Sun and Kato, 2016; Long et al., 2017). Currently, surgery is the main therapy for localized CRC, and adjuvant chemotherapy is also used for many patients.

Some markers have been shown to have prognostic or predictive value for colon cancer patients, meaning they can contribute to making therapeutic decisions with greater precision for specific patients. For example, Ki-67 and p53 have potential prognostic value in Dukes’ B and C colon cancer (Allegra et al., 2003), *KRAS* and *BRAF* have potential prognostic value in stage II and III resected colon cancer (Roth et al., 2010), the *BRAF* V600E mutation has been shown to be an independent prognostic factor for survival in stage II and stage III colon cancer patients (Farina-Sarasqueta et al., 2010), and *SNORA42* may be a prognostic biomarker in CRC (Okugawa et al., 2017). Indeed, screening potential prognostic biomarkers from multiple molecular levels is a crucial step for cancer therapy. Mounting evidence has indicated that miRNAs contribute to multiple pathophysiological processes and may be potential biomarkers for cancer diagnostics and therapy (Tong and Nemunaitis, 2008; Valihrach et al., 2019). Another type of non-coding RNA (ncRNA), lncRNAs, are implicated in diverse biological processes, especially epigenetic regulation (Lee, 2012; Morlando and Fatica, 2018). For example, miR-425-5p may be a potential prognostic biomarker for cervical cancer (Sun et al., 2017), and miRNA-based prognostic biomarkers have been found in other cancers, such as in pancreatic cancer (Guo et al., 2018). Integrating prognostic biomarkers of multiple classes, such as mRNA-miRNA-DNA methylation (Robles et al., 2015) and lncRNA-miRNA-mRNA (Zhang et al., 2018; Wang et al., 2019) can provide more references for cancer prognosis based on the potential cross-talk between the different molecular subtypes.

**FIGURE 1:**
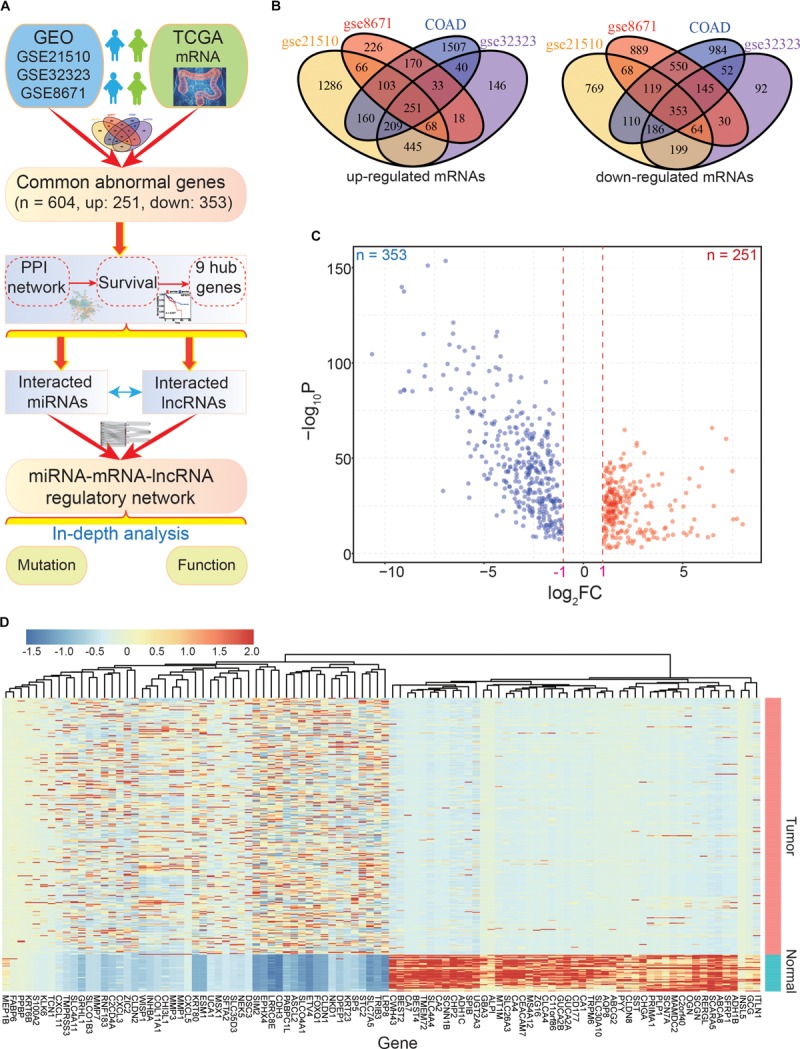
The main flow-chart and screening for differentially expressed genes. **(A)** The main flow-chart of this study. **(B)** The Venn diagrams of differentially expressed genes across different datasets. **(C)** The scatter-plot shows the distribution of dysregulated genes based on TCGA data. **(D)** The heatmap shows the detailed expression patterns of these genes in tumor and normal samples.

Additionally, recent studies have shown that many lncRNAs can compete with mRNAs for binding to miRNAs, acting as potential ceRNAs, which contribute to disease development (Cesana et al., 2011; Tan et al., 2015; Lu et al., 2016). The potential cross-talk between miRNA-lncRNA-mRNA has been widely studied (Qian et al., 2018; Chen Y. et al., 2019), and ceRNA network analysis has proven to be an effective method of screening potential prognostic biomarkers in diverse cancer types (Liu Z. et al., 2019; Song et al., 2019; Yao et al., 2019). Herein, we aimed to screen and identify potential prognostic biomarkers in CRC by constructing and analyzing a ceRNA network that was based on the integrative analysis of multiple genomics datasets (Figure 1A). According to potential relationships among diverse RNAs, we screened out a potential biomarker for cancer prognosis, the miR-200b-3p-MFAP5-AC005154.6 axis, which could potentially provide prognostic or predictive information for CRC patients.

## Materials and Methods

### Data Sources

High-throughput sequencing data of diverse cancers were obtained from TCGA^1^ using the “TCGAbiolinks” package (Colaprico et al., 2016)^2^. For CRC, there were over 500 individuals, including 480 tumor and 41 normal samples. To determine the DEGs common to different CRC samples, CRC datasets were also collected from GEO, including GSE32323 (Khamas et al., 2012) with 17 normal and 17 tumor samples, GSE21510 (Tsukamoto et al., 2011) with 44 normal and 104 tumor samples, and GSE8671 (Sabates-Bellver et al., 2007) with 32 normal and 32 tumor samples (Supplementary Table S1).

### Screening Potential Hub Genes

Based on TCGA and GEO datasets, the common DEGs were primarily identified using the criteria: | log_2_FC| > 1 and padj < 0.05. The screened genes were first queried for their biological function using The DAVID version 6.8 (Huang et al., 2009), and *z* scores (Walter et al., 2015) in biological processes of KEGG pathways were estimated using the following formula:

$$z\,\mathrm{score}=\frac{\left(u\,p-d\,o\,w\,n\right)}{\sqrt{c\,o\,u\,n\,t}}$$

where up and down were the numbers of up- and down-regulated genes, respectively, and count was the total number of involved genes.

To understand the potential function of these DEGs in CRC physiology, we also analyzed their contribution to the hallmarks of cancer (Subramanian et al., 2005)^3^, CGC (Futreal et al., 2004)^4^, core essential genes (obtained from the common genes of Hart et al. (2015); Blomen et al. (2015), and Wang et al. (2015)), and oncogenes and TSGs (Vogelstein et al., 2013). To understand the detailed expression patterns of the relevant genes, their distribution in KEGG pathways were also queried, and significantly enriched pathways were further presented using Pathview (Luo and Brouwer, 2013; Luo et al., 2017).

To survey the potential hub genes in CRC, PPI networks were constructed based on the DEGs using the STRING database^5^ with default parameters (Szklarczyk et al., 2019). PPI networks were constructed with up- and down-regulated genes. For the up-regulation PPI network, the key candidate genes were first screened based on two potential modules using the MCODE plug-in in Cytoscape 3.7.1 (Shannon et al., 2003). For the down-regulation PPI network, the CytoHubba plug-in in Cytoscape was used to display the top 100 potential hub genes according to node degrees.

### Potential Prognostic Values of Candidate Genes

It was important to next validate the potential prognostic values of the screened hub genes in CRC. Survival analyses were used to estimate the correlations of the candidate genes (including candidate miRNAs and lncRNAs) with prognoses. The clinical data of CRC cases, including survival status, cancer stage and grade, survival time, and molecular subtype, were obtained from TCGA using the “TCGAbiolinks” package (Colaprico et al., 2016). The log-rank test was used to estimate potential differences, and statistical significance was set at *p* < 0.05. Furthermore, prognostic results for candidate genes were also obtained from the GEPIA^6^ (Tang et al., 2017) and StarBase (Li et al., 2014) databases.

### Screening Related miRNAs and lncRNAs Based on the Hub mRNAs

Candidate hub mRNAs with potential prognostic value in CRC were used to screen related miRNAs based on biological interactions. The miRNA:mRNA interactions were first collected from StarBase (Li et al., 2014), and then were further filtered based on their prognostic results. Next, the selected miRNAs were used to survey associated lncRNAs from StarBase (Li et al., 2014) and miRNet (Fan and Xia, 2018). The identified lncRNAs were further analyzed for significant correlations with cancer prognosis.

### Construction and In-Depth Analysis of the ceRNA Network

The screened hub genes, interacting miRNAs, and associated lncRNAs, were constructed into a ceRNA network based on their regulatory relationships using the R package of “networkD3”^7^.

According to the constructed primary ceRNA network, an in-depth analysis was performed for the involved RNAs, despite all of them being potential prognostic markers. The miRNA-mRNA interactions were further estimated based on their expression patterns and correlations, and detailed isomiR expression patterns were investigated because of the phenomenon of multiple isomiRs in the miRNA world. An increasing number of studies have shown that the small ncRNAs are not single sequences, but contain a series of multiple sequences with diverse expression patterns (Morin et al., 2008; Neilsen et al., 2012; Tan et al., 2014; Guo and Liang, 2018; Desvignes et al., 2020). Indeed, these multiple isomiRs are regulatory molecules in coding-non-coding RNA networks; therefore, it is necessary to discuss the detailed expression of isomiRs in this study. Moreover, a dysregulated expression pattern was also an important factor to identify RNAs as potential prognostic markers, and the final screened RNAs were queried for their expression patterns across diverse cancer types.

### Statistical Analysis and Network Visualization

Differentially expressed gene profiles were estimated using DESeq2 (Love et al., 2014), and hypothesis testing in relevant analyses, mainly including a Wilcoxon rank-sum test, a Kruskal–Wallis test, and a paired *t*-test, were used to estimate the potential differences between groups. Interactions between different RNAs, PPI networks, and coding-non-coding RNA regulatory networks were visualized using Cytoscape 3.7.1 (Shannon et al., 2003). Venn distributions were generated using a publicly available tool^8^. All statistical analyses were analyzed using the R programming language (version 3.6.1).

## Results

### Survey of the Common Expression Landscape in CRC

To identify the common DEGs in CRC with higher confidence levels, we performed an integrative analysis of GEO and TCGA datasets to find the commonly dysregulated genes (Figure 1A). Based on the four CRC datasets, a total of 604 common dysregulated mRNAs, including 251 up- and 353 down-regulated transcripts, were obtained (Figures 1B–D). Indeed, most of the DEGs were found in two or three datasets, and some were detected in a specific dataset. Based on the detailed expression patterns in TCGA, these DEGs were significantly divergent between normal and tumor samples (Figure 1D).

### Functional Analysis of the DEGs

**FIGURE 2:**
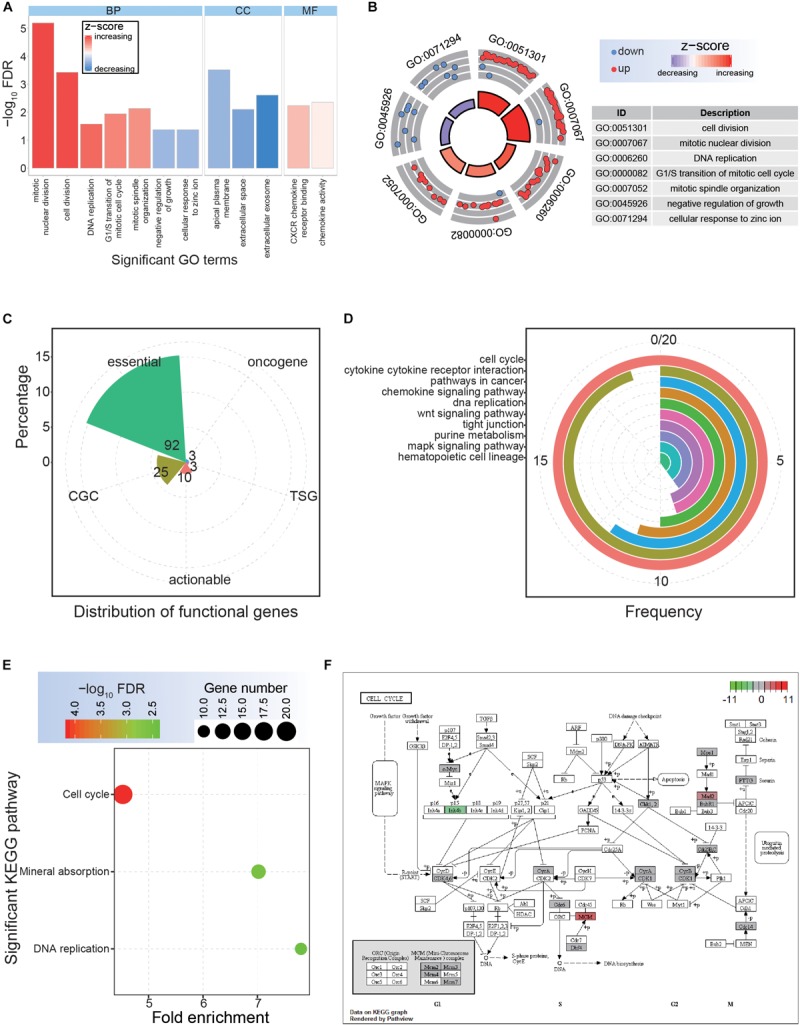
Functional analysis of the common dysregulated genes. **(A)** The distributions of significantly enriched Gene Ontology (GO) terms. BP, biological process; CC, cellular component; MF, molecular function. **(B)** Significantly enriched GO terms for biological process. **(C)** The detailed distributions of functional genes. **(D)** The detailed gene distributions of involved Kyoto Encyclopedia of Genes and Genomes (KEGG) pathways. **(E)** Significantly enriched KEGG pathways. **(F)** The expression patterns of crucial cell cycle genes.

Although a series of common DEGs in CRC were collected based on multiple datasets, their potential functional implications remained unknown. Therefore, it was necessary to next investigate their potential cellular functions, as this would contribute to surveying the potential hub genes. For these common DEGs, significant GO terms and KEGG pathways were enriched (Figure 2). Specifically, seven significant biological processes terms were enriched, including mitotic nuclear division, cell division, and DNA replication (Figures 2A,B), indicating potential roles of these DEGs in tumorigenesis. Among these DEGs, 92 were identified as core essential genes, and 25 were CGC genes (Figure 2C), suggesting critical roles in cancer-associated pathways. The cell cycle pathway was the most common pathway among the involved genes and was also an enriched KEGG pathway *via* DAVID analysis (Figures 2D,E). Further analysis based on the cell cycle pathway in CRC showed crucial genes with abnormal expression patterns (Figure 2F), implying an important role for these abnormally expressed genes in the occurrence and development of CRC.

### Screening Hub Genes Based on PPI Interaction Networks and Potential Value to Cancer Prognosis

**FIGURE 3:**
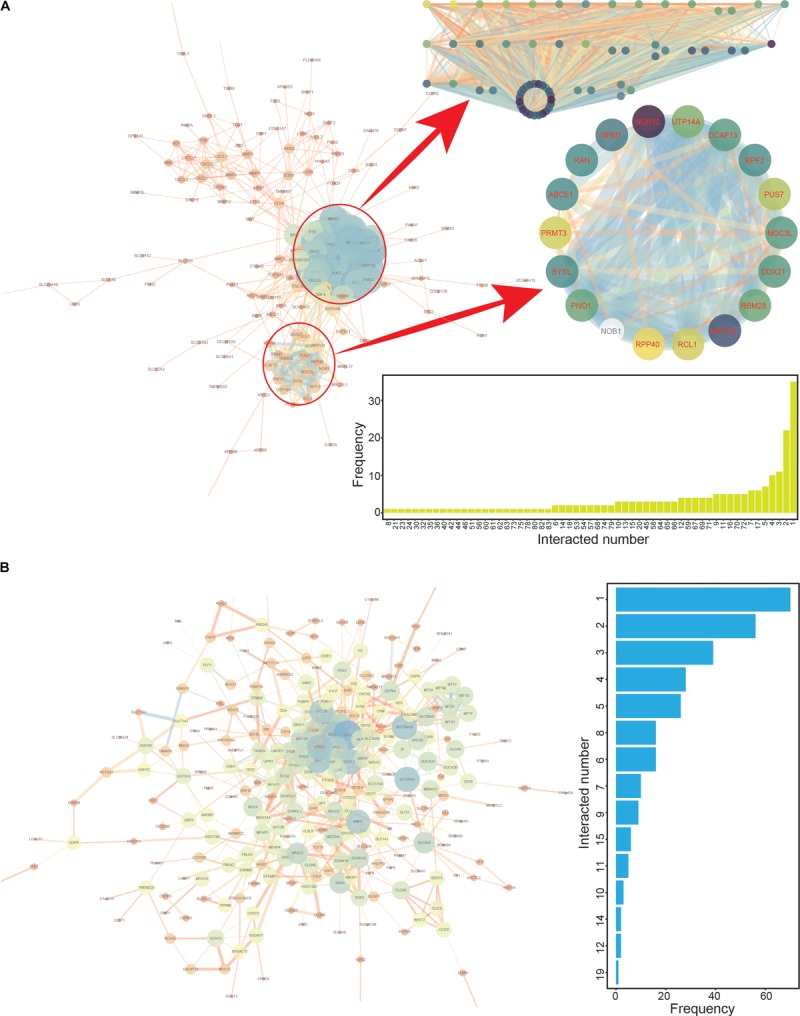
The protein-protein interaction (PPI) networks constructed based on up- and down-regulated genes. **(A)** The PPI interaction network based on up-regulated genes, with two specific modules highlighted. The numbers of interacting genes are also presented. **(B)** The PPI interaction network based on down-regulated genes. The numbers of interacting genes are also presented.

To screen the CRC hub genes, PPI networks were constructed based on the common up- and down-regulated genes, respectively (Figure 3). The two modules were enriched in the network of up-regulated genes (Figure 3A), and these involved genes (module 1 contained 64 genes and module 2 contained 18 genes, Supplementary Tables S2, S3) that might be candidate hub genes worthy of performing further analysis regarding cancer prognosis. Simultaneously, the top 100 genes (≥4 interactions with other genes) with the highest scores were the potential candidate hub genes based on the network of down-regulated genes (Supplementary Table S4 and Figure 3B). Thus, a total of 182 candidate hub genes were collected from PPI interaction networks, and these genes were further investigated in survival analyses to identify potential prognostic correlations with these genes.

**FIGURE 4:**
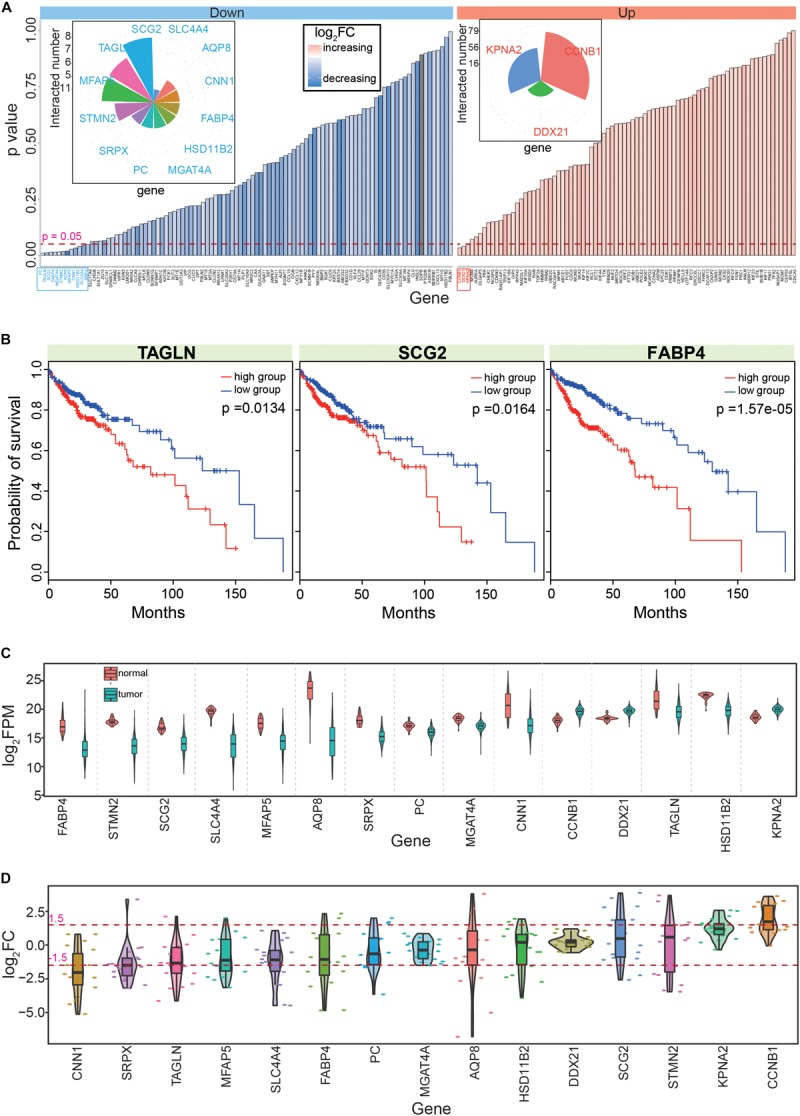
Screening the 15 candidate genes according to PPI networks and survival analyses. **(A)** Survival analyses of the 182 genes that are obtained from the PPI network; distributions of *p* values and their detailed fold change values are presented. The detailed numbers of significant gene interactions are also presented. Down shows down-regulated genes, up shows up-regulated genes, and log_2_FC shows fold change of each gene in tumor samples. Genes with significant correlations with cancer prognosis are highlighted in blue or red. **(B)** Examples of genes with significant correlations with CRC prognosis. **(C)** Expression patterns of the 15 screened hub genes. Normal means normal (non-cancerous) samples, and tumor means tumor samples. **(D)** Distributions of the abnormal expression patterns of the 15 hub genes via a pan-cancer analysis.

Based on survival analyses of these primarily screened candidate hub genes in CRC, we obtained 15 hub genes (Supplementary Table S5, including 12 down- and three up-regulated genes) with significant correlations to prognosis (Figures 4A,B). Among these genes, most had multiple interactions with other genes, suggesting important roles in biological processes as hub genes. These 15 candidate genes were significantly dysregulated in CRC (Figure 4C), and most showed consistent expression patterns across many cancers, although some showed the opposite expression patterns in different tissues (Figure 4D). For example, AQP8 showed various expression patterns in diverse tissues, suggesting specific roles for AQP8 in different cancers. Indeed, AQP8 may inhibit colorectal cancer growth and metastasis by decreasing PI3K/AKT signaling and PCDH7 expression (Wu Q. et al., 2018); thus, AQP8 status in colorectal carcinoma has a potential clinical significance (Wang et al., 2012).

Next, these 15 hub genes were queried for correlations with cancer prognosis in the GEPIA database, which revealed that nine had consistent survival results. Thus, these nine genes (AQP8, CCNB1, CNN1, FABP4, KPNA2, MFAP5, PC, SCG2, and TAGLN) were selected as the final hub genes in CRC. Among them, most have been previously reported to be crucial genes for CRC tumorigenesis. For example, the HnRNPR-CCNB1/CENPF axis may contribute to gastric cancer proliferation and metastasis (Chen E. B. et al., 2019), CNN1 may be a potential prognostic marker of bladder cancer according to a bioinformatics analysis (Liu Y. et al., 2019), and the combined detection of CEA with FABP4 and FABP6 may improve the diagnostic efficacy of CRC (Zhang et al., 2019). These previous findings indicated that these nine hub genes have important roles in the occurrence and development of CRC, which suggested that they could also be potential prognostic markers.

### Screening Related miRNAs Based on the Nine Hub Genes

**FIGURE 5:**
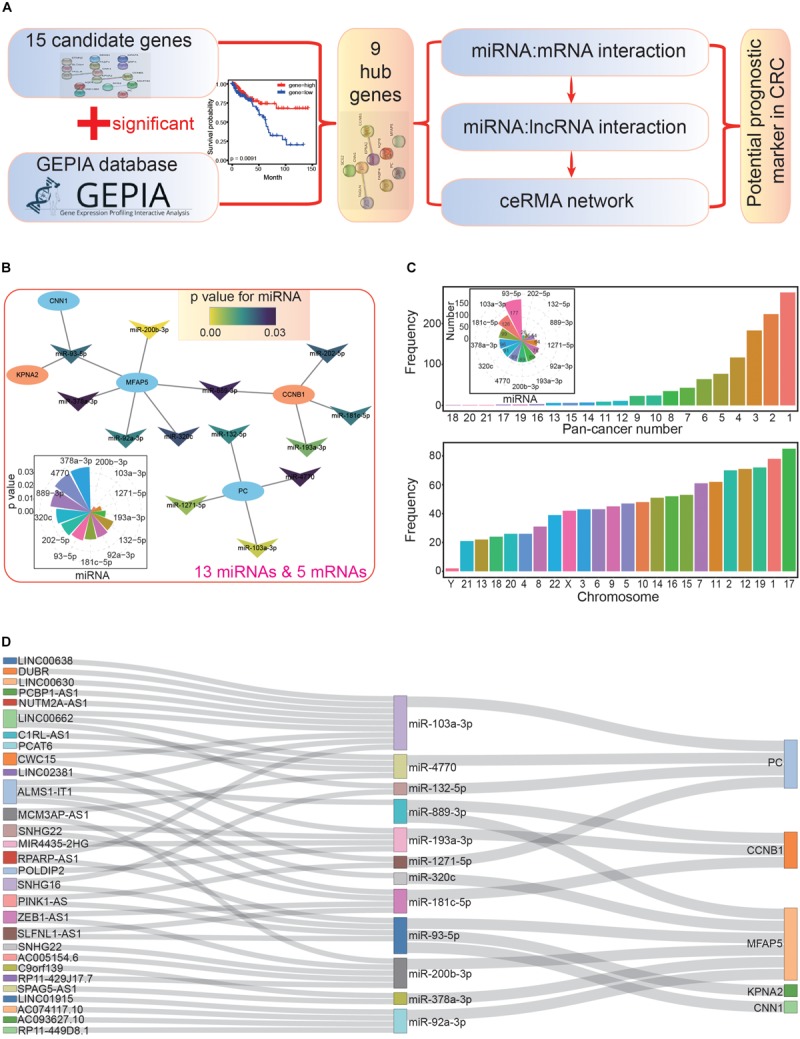
Construction of the primary competing endogenous RNA (ceRNA) network. **(A)** The flowchart for further screening and analysis of the ceRNA network based on candidate hub genes. **(B)** The miRNA-mRNA interaction network. All miRNAs have significant correlations in the survival analysis (*p* < 0.05); the distribution of *p* values is also presented. **(C)** The picture above shows the distributions of miRNA-lncRNA interactions, many of which are validated in diverse cancer types (pan-cancer number). The number of lncRNAs that interacted with each miRNA are also presented. The picture below shows the chromosomal location of the involved lncRNAs. **(D)** The primary constructed ceRNA network. All involved RNAs are associated with prognosis.

Next, we used the nine hub genes to further survey miRNAs and lncRNAs that interacted with them to construct the ceRNA network (Figure 5A). miRNAs related to the hub genes were collected according to validated miRNA:mRNA interactions. These candidate miRNAs were further investigated in survival analyses to understand their potential values in cancer prognosis. Through this, we obtained 13 miRNAs with potential value as prognostic markers, and these miRNAs could interact with five hub genes (Supplementary Table S6 and Figure 5B). All 13 miRNAs negatively regulated the hub genes by controlling the enrichment levels of these genes. Indeed, many studies have shown that miRNAs have crucial biological functions in CRC. For example, miR-92a and miR-144^∗^ may be potential biomarkers of non-invasive colorectal cancer (Choi et al., 2019), and miR-103a may be a new regulator of Wnt signaling as an onco-miRNA (Fasihi et al., 2018). In the miRNA-mRNA interaction network, we found that MFAP5 had the most interactions with miRNAs (it could be regulated by six miRNAs), while KPNA2 and CNN1 only interacted with one specific miRNA, miR-93-5p (Figure 5B). These interactions represented regulatory relationships in the coding-non-coding RNA network, and multiple interactions for specific genes suggested a complex regulatory network.

### Screening Associated lncRNAs Based on the 13 Candidate miRNAs

Based on these 13 miRNAs (herein, miRNAs were treated as intermediaries to connect mRNAs and lncRNAs), we further obtained a series of lncRNAs with possible interactions. Most of these interactions were validated in multiple cancer types (above picture in Figure 5C), indicating that these miRNA-lncRNA interactions existed in diverse tissues. Among the miRNAs, miR-93-5p had the most interacting lncRNAs (177), followed by miR-103a-3p (126), and miR-181c-5p (99). Furthermore, we also analyzed the distributions of these lncRNAs, and chromosome 17 contained the most, followed by chromosomes 1 and 19 (below picture in Figure 5C). To understand the potential prognostic values of these lncRNAs, we performed survival analyses, after which, we collected 29 lncRNAs that interacted with 12 miRNAs, all of which had potential prognostic value.

### Construction of the ceRNA Network and Further Analysis at the isomiR Level

According to the screened hub genes, their interacting miRNAs, and associated lncRNAs, a ceRNA network was constructed based on their biological interactions. Although each step might involve a large number of candidate mRNAs, miRNA, or lncRNAs, only several of them were used to construct the ceRNA network after a step-by-step screening process, especially for simultaneously screening interactions and significant correlations with cancer prognosis. Ultimately, five hub genes (PC, CCNB1, MFAP5, KPNA2, and CNN1), 12 related miRNAs, and 29 associated lncRNAs were used to construct the ceRNA network (Figure 5D).

**FIGURE 6:**
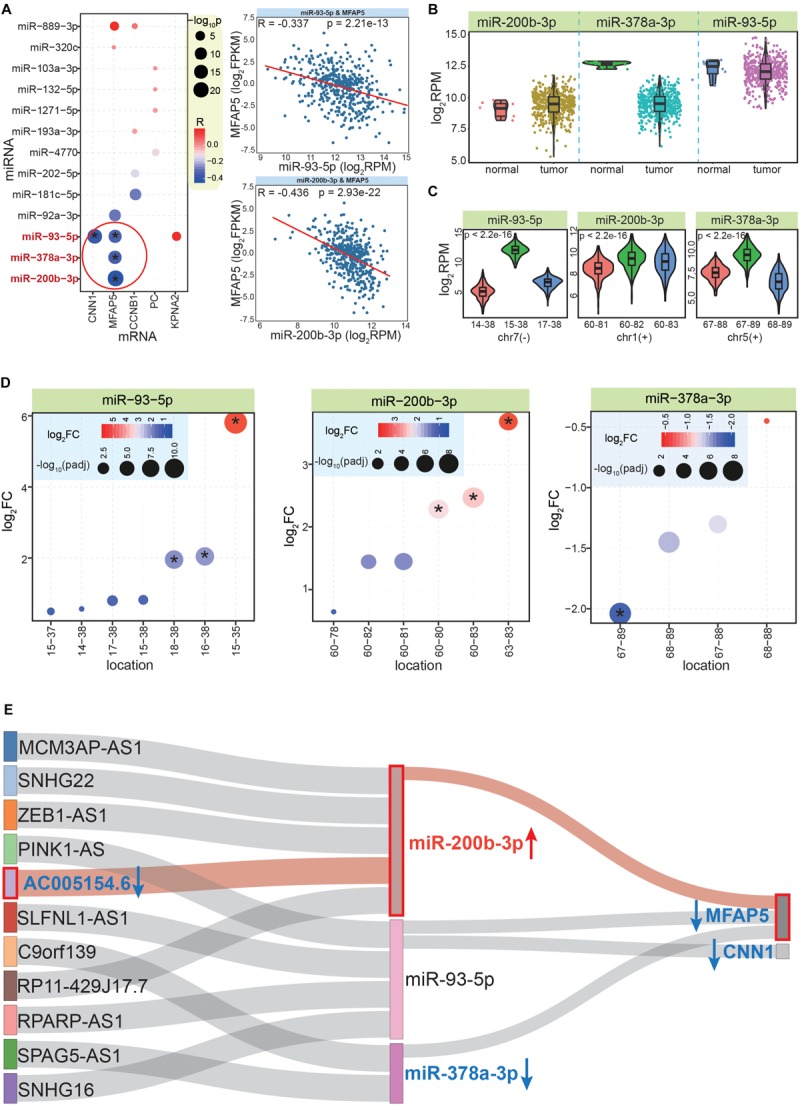
Further screening and identifying crucial ceRNA network. **(A)** The bubble plot shows co-expression correlations between mRNAs and related miRNAs; * indicates a significant negative correlation between the miRNA and mRNA pair. The images to the right show examples of the detailed expression patterns of the miRNAs and mRNAs. **(B)** The expression patterns between normal and CRC tumor samples for the three screened miRNAs. **(C)** Expression patterns of the most dominant three isomiRs in each miRNA locus; *p* values are estimated based on the Kruskal–Wallis test. The horizontal ordinate is the relative start and end point of each isomiR. **(D)** Differential expression patterns of multiple isomiRs in each miRNA locus. * indicates significantly abnormally expressed isomiRs (| log_2_FC| >1.5 and padj < 0.05). **(E)** Further analysis to identify crucial interactions in the ceRNA network, which reveals that the MFAP5-miR-200b-3p-AC005154.6 axis is a potential prognostic biomarker for CRC. Blue arrow shows decreased expression in CRC, red arrow shows increased expression. The MFAP5-miR-200b-3p-AC005154.6 axis is highlighted in pink.

Although only validated miRNA:mRNA interactions were analyzed, it was necessary to determine whether there were negative correlations between them, as this would indicate that these interactions exist in CRC. According to StarBase (Li et al., 2014), there were four pairs of miRNA-mRNA interactions with significant negative correlations, including miR-93-5p:CNN1, miR-378-3p:MFAP5, miR-93-5p:MFAP5, and miR-200b-3p:MFAP5 (Figure 6A). These pairs contained only three miRNAs and two mRNAs, among which MFAP5 was the gene with the most (three) miRNA interactions. The expression patterns of these three miRNAs showed that miR-200b-3p was significantly up-regulated, while miR-378a-3p was significantly down-regulated (Figure 6B). This highlighted that examining expression patterns was a crucial step to further screen correlated RNAs based on ceRNA network.

Because of the multiple isomiRs within a single miRNA locus, we also analyzed the detailed expression patterns at the isomiR level for to each miRNA locus. Interestingly, we found there were different expression patterns for the top three dominantly expressed isomiRs of these miRNA loci (Figure 6C). For miR-93-5p, the dominant isomiR had a distinctly increased expression compared with the others, while the other miRNA loci showed closer expression patterns of the dominant isomiRs, especially the miR-200b-3p locus (Figure 6C). The non-random expression distributions suggested that multiple isomiRs might be strictly regulated, which may contribute to maintaining miRNA:mRNA interactions that negatively regulate mRNA expression. Indeed, due to diverse sequences and enrichment levels, these isomiRs also had various expression patterns in cancer (Figure 6D). Some isomiRs were identified as abnormally expressed sequences, while others were normally expressed in tumor tissues (despite these diverse isomiRs were generated from a specific miRNA locus). For example, in the miR-378-3p locus, one specific isomiR was significantly dysregulated, while others did not show altered expression patterns (Figure 6D).

### Screening Potential Prognostic Markers and Further in-Depth Analysis

We next filtered candidate RNAs according to miRNA:mRNA interactions and co-expression analysis. Thus, a further ceRNA network was constructed that contained two mRNAs (MFAP5 and CNN1), three miRNAs, and 11 lncRNAs (Supplementary Table S7 and Figure 6E). In this interaction network of three RNA classes, because all of the included molecules were significantly correlated with cancer prognosis, their expression patterns were important factors to screen for potential prognostic markers. Thus, the 11 lncRNAs were further analyzed for their expression patterns in CRC. Finally, we found that AC005154.6 had a consistent expression pattern with MFAP5 (significantly down-regulated in CRC), and both had opposing expression patterns with the mediating miRNA, miR-200b-3p (Figure 6E).

**FIGURE 7:**
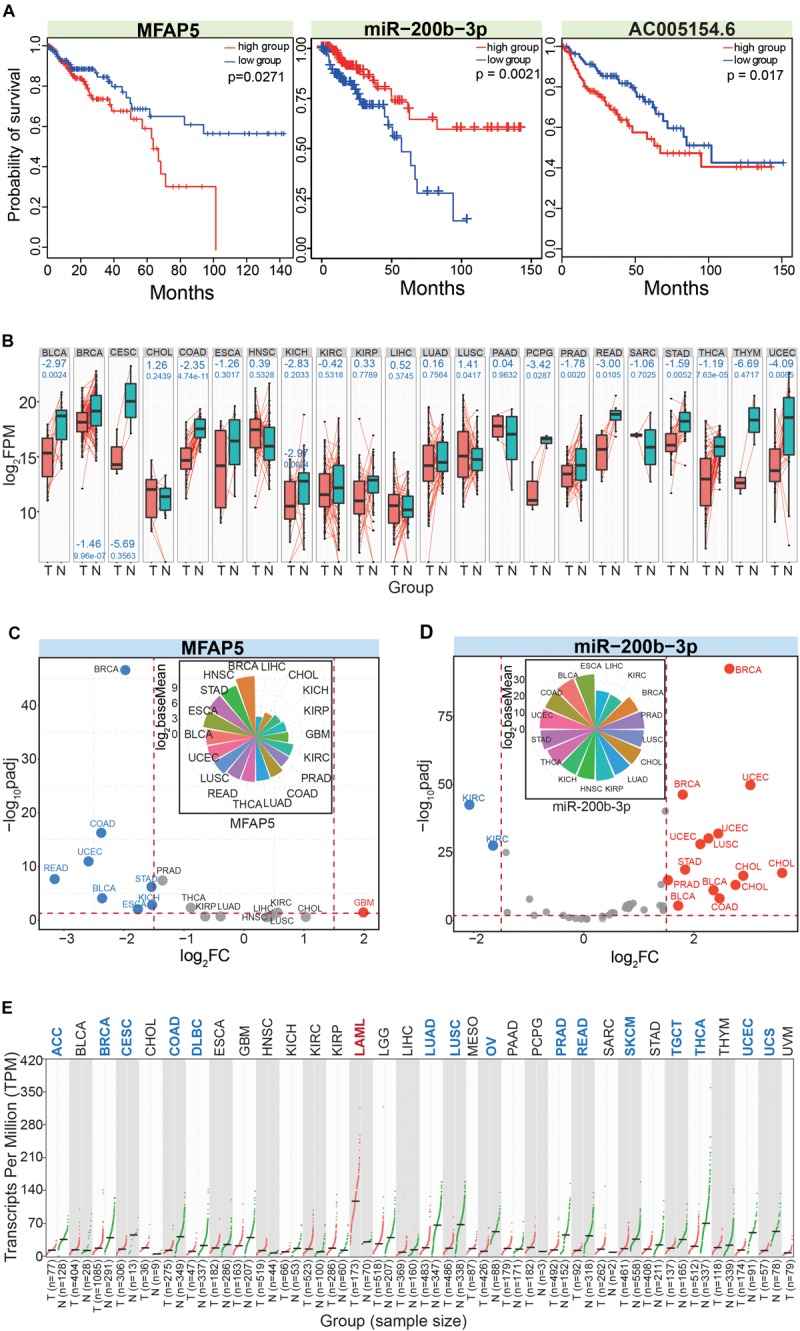
In-depth analysis of the screened RNAs. **(A)** Survival curves for the involved RNAs show their potential values as prognostic markers. **(B)** Expression patterns of MFAP5 based on paired analysis across different cancer types. The log_2_FC (above blue number) and *p* (below blue number) values are presented in relevant images. T, tumor samples; N, normal samples. **(C)** The scatter plot shows expression patterns of MFAP5 based on the DeSeq algorithm. The picture above shows distributions of baseMean values. **(D)** The scatter plot shows expression patterns of miR-200b-3p based on the DeSeq algorithm. The picture above shows distributions of baseMean values. Here, the three dominant isomiRs in the miR-200b-3p locus are simultaneously analyzed. **(E)** Expression pattern of AC005154.6 based on Gene Expression Profiling Interactive Analysis (GEPIA).

We finally obtained MFAP5, miR-200b-3p, and AC005154.6 as potential prognostic markers in CRC. All three had potential prognostic value in CRC, and MFAP5 and AC005154.6 had consistent trends (Figure 7A). To further understand these three RNAs at their different molecular levels, they were queried for their detailed expression patterns in other cancer types. Based on paired samples, MFAP5 showed consistent expression patterns across different tissues, and most showed significant down-regulation (Figure 7B). Similar results were found in all samples (Figure 7C), and the relative expression patterns via pan-cancer analysis showed that MFAP5 was an important gene in tumorigenesis. Indeed, MFAP5 (microfibril-associated protein 5) may facilitate the distinction between pseudo-invasive and true-invasive lesions among colonic adenomatous polyps (Zhao et al., 2019). MFAP5 blockade can inhibit fibrosis and enhance chemosensitivity in ovarian and pancreatic cancer (Yeung et al., 2019), and can promote basal-like breast cancer progression by activating epithelial-to-mesenchymal transition (Wu et al., 2019).

As a potentially crucial miRNA that interacted with MFAP5, miR-200b-3p also showed relatively consistent expression across diverse cancer types (Figure 7D). We analyzed the three dominantly expressed isomiRs of the miR-200b-3p locus to track the expression patterns of these ncRNAs. The three isomiRs always showed consistent expression in specific tissues despite having heterogeneous sequences and expression, indicating potential collaborative relationships among these multiple isomiRs in the coding-non-coding RNA regulatory network. miR-200 has been validated as an important regulatory molecule. Specifically, the miR-200b-3p/p38IP pair regulates monocyte/macrophage differentiation (Yu et al., 2016), miR-200b-3p in plasma may be a potential diagnostic biomarker of oral squamous cell carcinoma (Sun et al., 2018), and quercetin-induced miR-200b-3p can regulate the mode of self-renewal divisions in pancreatic cancer (Nwaeburu et al., 2017). Thus, miR-200 has an important role in the pathological processes of multiple cancers, and diverse isomiRs, especially the dominantly expressed isomiRs, largely contribute to synergistically regulating these pro-tumor processes. For the lncRNA AC005154.6, most cancer types showed down-regulated expression (Figure 7E), and these consistent expression patterns suggested it played a role in tumorigenesis. AC005154.6 has been reported to be highly expressed in the pituitary (Levran et al., 2018), but details of its biological functions are unknown.

## Discussion

Identifying new prognostic biomarkers is essential for CRC, as this also contributes to exploring the mechanisms of metastasis and surveying candidate gene targets for therapy. Herein, according to the ceRNA hypothesis, we proposed an approach to construct a ceRNA network in CRC based on multiple RNA datasets. First, the common dysregulated mRNA profiles were filtered across several sequencing datasets, and the significant DEGs were further queried for their potential functional implications. Second, potential hub genes were screened from PPI networks, and candidate hub genes were screened based on their prognostic value in CRC. Primarily screened hub genes were further validated based on their expression patterns across diverse cancer types, which revealed their expression trends and specificities in different tissues. Third, candidate hub genes were extended to interacting miRNAs because these small ncRNAs are important negative regulators in coding-non-coding RNA networks. The miRNA:mRNA interactions were validated based on their biological and expression relationships. Then, candidate miRNAs were screened by survival analyses to estimate their prognostic value. Finally, based on the screened miRNAs from the hub genes, relevant lncRNAs were collected and queried for their potential as prognostic markers. Thus, we screened hub genes, and relevant miRNAs and lncRNAs that were associated with CRC prognosis, as any of them may be a potential clinical biomarker. These obtained RNAs also have potential biological relationships and close expression correlations, so they were used to construct a ceRNA network to further screen candidate prognostic CRC biomarkers. Finally, the dysregulated expression patterns were simultaneously analyzed to predict possible regulatory relationships among the different identified RNAs. Furthermore, the expression patterns of miRNAs were dissected at the isomiR level. Thus, a mRNA-miRNA-lncRNA axis could be identified as a potential prognostic marker, and the involved RNAs were analyzed in-depth across diverse cancer types to identify trends in their expression.

In this study, miRNAs were used to link mRNAs and lncRNAs, which is an important step when constructing a ceRNA network. Given tumor heterogeneity and the multitude of variables that influence clinical progress, the combination of multiple RNAs provides a more comprehensive prognostic analysis. Indeed, many previous studies have found great prospects for the regulation of CRCs by lncRNAs, miRNAs and mRNAs (Marisa et al., 2013; Pizzini et al., 2013; Zhou et al., 2016). Aberrant lncRNA expression is associated with tumorigenesis, tumor progression, and metastasis (Shi et al., 2013). Although only a few lncRNAs have been investigated in CRC, existing results have demonstrated that lncRNAs may be ideal prognostic biomarkers for this malignancy (Smolle et al., 2014). In the integrative analysis of the different RNA classes, we also analyzed multiple isomiRs within the different miRNA loci. Diverse isomiRs expand miRNA:mRNA interactions, and the widespread interactions between small ncRNAs and mRNAs contribute to flexible regulation and regulatory effectiveness. Multiple isomiRs provide a synergistic regulatory pattern among diverse small ncRNAs, although these isomiRs usually show expression and sequence heterogeneities. Similar to homologous and/or clustered classical miRNAs, the phenomenon of isomiRs enriches and complicates studying small regulatory RNAs.

Based on first screening hub genes and then identifying relevant miRNAs followed by lncRNAs, all of the candidate RNAs are potential prognostic markers in CRC at the individual molecular level. Based on the potential biological and expression relationships, especially for expression patterns of isomiRs within the miRNA loci, we further screened the MFAP5-miR-200b-3p-AC005154.6 axis, which may be a potential prognostic biomarker for CRC. MFAP5 promotes tumor progression and bone metastasis in several cancers (Leung et al., 2014; Wu Z. et al., 2018), and miR-200b-3p negatively regulates MFAP5 expression; finally, AC005154.6 interacts with miRNA-200b-3p. Thus, the interactions among these RNAs may further contribute to relevant biological pathways and CRC pathophysiology. Although previous studies have shown that these RNAs have important roles in multiple biological processes, especially for MFAP5 and miR-200b-3p in tumorigenesis, more studies are needed that are focused on the mechanistic interactions between MFAP5, miR-200b-3p and AC005154.6, especially regarding their functional relationships in the occurrence and progression of CRC.

## Data Availability Statement

The datasets generated for this study can be found in the manuscript.

## Author Contributions

LG and TL designed this study. LG, GY, YK, SL, RD, LS, WJ, BQ, and ZY participated in data analysis. LG and TL wrote the manuscript. All authors read and accepted the final version. TL was responsible for coordinating and supervising the entire project.

## Conflict of Interest

The authors declare that the research was conducted in the absence of any commercial or financial relationships that could be construed as a potential conflict of interest.

## Footnotes

1 https://tcga-data.nci.nih.gov/tcga/

2 http://doi.org/10.1093/nar/gkv1507

3 http://software.broadinstitute.org/gsea/msigdb/

4 http://cancer.sanger.ac.uk/census

5 http://string-db.org/

6 http://gepia.cancer-pku.cn/detail.php

7 https://CRAN.R-project.org/package=networkD3

8 http://bioinformatics.psb.ugent.be/webtools/Venn/

## Abbreviations

ACC: adrenocortical carcinoma
BLCA: bladder urothelial carcinoma
BP: biological process
BRCA: breast invasive carcinoma
CC: cellular component
ceRNAs: competitive endogenous RNAs
CESC: Cervical squamous cell carcinoma and endocervical adenocarcinoma
CGC: cancer gene census
CHOL: cholangiocarcinoma
COAD: colon adenocarcinoma
CRC: colorectal cancer
DAVID, Database for Annotation: Visualization and Integrated Discovery
DEG: differentially expressed gene
DLBC: lymphoid neoplasm diffuse large B-cell lymphoma
ESCA: esophageal carcinoma
GBM: glioblastoma multiforme
GEO: Gene Expression Omnibus
GEPIA: Gene Expression Profiling Interactive Analysis
GO: gene ontology
HNSC: head and neck squamous cell carcinoma
KEGG: Kyoto Encyclopedia of Genes and Genomes
KICH: kidney chromophobe
KIRC: Kidney renal clear cell carcinoma
KIRP: kidney renal papillary cell carcinoma
LAML: acute myeloid leukemia
LGG: brain Lower grade glioma
LIHC: liver hepatocellular carcinoma
lncRNAs: long non-coding RNAs
LUAD: lung adenocarcinoma
LUSC: lung squamous cell carcinoma
MESO: Mesothelioma
MF: molecular function
miRNAs: microRNAs
OV: ovarian serous cystadenocarcinoma
PAAD: pancreatic adenocarcinoma
PCPG: pheochromocytoma and paraganglioma
PPI: protein-protein interaction
PRAD: prostate adenocarcinoma
READ: rectum adenocarcinoma
SARC: sarcoma
SKCM: skin cutaneous melanoma
STAD: stomach adenocarcinoma
STRING: Search Tool for the Retrieval of Interacting Genes
TCGA: The Cancer Genome Atlas
TGCT: testicular germ cell tumors
THCA: thyroid carcinoma
THYM: thymoma
TSG: tumor suppressor gene
UCEC: uterine corpus endometrial carcinoma
UCS: uterine carcinosarcoma
UVM: uveal melanoma.
